# Tunable Strong Plasmon-Exciton Coupling in a Low-Loss Nanocuboid Dimer with Monolayer WS_2_

**DOI:** 10.3390/nano15191497

**Published:** 2025-09-30

**Authors:** Fan Wu, Zhao Chen

**Affiliations:** 1 College of Science, Beijing Forestry University, Beijing 100083, China; 2 College of Mathematics and Physics, Beijing University of Chemical Technology, Beijing 100029, China

**Keywords:** strong plasmon–exciton coupling, silver nanocuboid dimer, subradiant plasmonic modes, two-dimensional materials, coupling control

## Abstract

Strong coupling between plasmons and excitons in two-dimensional materials offers a powerful route for manipulating light–matter interactions at the nanoscale, with potential applications in quantum optics, nanophotonics, and polaritonic devices. Here, we design and numerically investigate a low-loss coupling platform composed of a silver nanocuboid dimer and monolayer of WS_2_ using finite-difference time-domain (FDTD) simulations. The dimer supports a subradiant bonding plasmonic mode with a linewidth as narrow as 60 meV. This ultralow-loss feature enables strong coupling with monolayer WS_2_ at relatively low coupling strengths. FDTD simulations combined with the coupled oscillator model reveal a Rabi splitting of ~60 meV and characteristic anticrossing behavior in the dispersion relations. Importantly, we propose and demonstrate two independent tuning mechanisms—loss engineering through nanocuboid tilt and coupling-strength modulation through the number of WS_2_ layers—that enable transitions between weak and strong coupling regimes. This work provides a low-loss and tunable plasmonic platform for studying and controlling strong light–matter interactions in plasmon-two-dimensional material systems, with potential for room-temperature quantum and optoelectronic devices.

## 1. Introduction

Strong coupling between local cavity modes and quantum emitters is a key topic in quantum optics [1,2,3]. In this regime, the rate of coherent energy exchange between photons and excitons exceeds dissipation, giving rise to hybrid eigenstates-polaritons—that combine photonic and excitonic characteristics. Such hybrid states underpin emerging applications in low-threshold lasing [2,4], quantum information processing [5,6,7], and quantum networks [8]. While solid-state optical microcavities have enabled landmark demonstrations of strong coupling [9,10], they often require cryogenic conditions and are constrained by the diffraction limit, limiting device scalability. Plasmonic nanostructures circumvent these challenges by concentrating electromagnetic fields into deep-subwavelength volumes, thereby enhancing light–matter interactions and enabling strong coupling under ambient conditions [11,12]. Many strong plasmon−exciton coupling systems have been implemented experimentally [13,14,15,16,17,18], and a series of unique optical properties such as chiral optical properties [19,20,21,22,23], nonlinear optical properties [24,25,26,27] of the plexcitonic systems have been revealed. These findings facilitate our understanding of optical interactions in the strong coupling regime and open up new possibilities for the application of nano-optics.

Transition-metal dichalcogenides (TMDCs), as a class of two-dimensional layered materials, have garnered significant attention over the past decade [28]. Their direct bandgap, high oscillator strength, and large exciton binding energy make them an ideal excitonic platform for exploring strong light–matter interactions [28,29,30]. Various plasmonic architectures have been explored to achieve strong coupling between plasmons and excitons in TMDCs [31,32,33,34,35,36]. Among these, metal nanoparticles are widely employed due to their ability to support localized surface plasmon resonances (LSPRs), which confine light to deep subwavelength volumes, thereby reducing the mode volume and enhancing light–matter interactions [34,35,37,38,39,40].

Nevertheless, the large plasmonic loss raises the critical coupling strength required to enter the strong coupling regime, imposing more stringent demands on the design of architectures, and reducing the coherence time [41,42,43,44]. Therefore, the development of low-loss plasmonic platforms remains of great importance. On the other hand, most existing approaches for tuning the coupling system have focused on modifying coupling strength control, such as adjusting the number of 2D material layers or varying the concentration of dye molecules to control the coupling strength [45,46,47,48,49]. According to the fundamental principles of strong coupling, however, regulating the system loss offers another important pathway for tailoring the coupling state—yet this aspect has been rarely reported.

Thus, a coupling system with tunable loss is expected to provide a new dimension for the regulation of plexciton systems.

Plasmonic dimers present a compelling solution to these challenges. The subradiant modes supported by dimer configurations can significantly suppress radiative losses while preserving a small mode volume [50,51,52]. In addition, the geometric degrees of freedom inherent to dimers provide multiple avenues for mode control, offering enhanced tunability and design flexibility for strongly coupled plexcitonic systems [53,54].

In this work, we employed FDTD simulations to construct a strong coupling system composed of a silver nanocuboid dimer (NCD) and monolayer of WS_2_. We first investigated the optical properties of the bonding and antibonding modes supported by the NCD. The subradiant bonding mode, benefiting from suppressed radiative loss, exhibits an ultranarrow linewidth of only 60 meV, making it an ideal platform for achieving strong light–matter interactions. This result exhibits only about one quarter of the loss of the dimer’s radiative mode (approximately 247 meV) that has previously been used to realize plasmon-TMDCs’ strong coupling [39,55]. Leveraging the exceptionally low loss of the bonding mode, we realized strong coupling between the NCD and monolayer WS_2_ at relatively modest coupling strengths. The coupled oscillator model (COM) was employed to analyze the absorption and dispersion spectra of the system. The theoretical predictions show excellent agreement with FDTD simulations, and the anticrossing behavior observed in the dispersion curves provides unambiguous evidence that the system operates in the strong coupling regime. Finally, we propose two complementary strategies for tuning the coupling state: (i) modulating the system loss by adjusting the tilt angle of the nanocuboid to switch between different coupling regimes, and (ii) tuning the coupling strength by varying the number of WS_2_ layers. Taken together, these two approaches enable control from both the dissipation and interaction strength perspectives, thus providing a more versatile and systematic route to engineer the coupled system and optimize its performance for targeted applications. Our work is expected to contribute to the advancement of research on strong plasmon–2D material coupling, providing new insights and strategies for controlling light–matter interactions in hybrid nanostructures.

## 2. Structural Design and Mode Analysis

**Figure 1:**
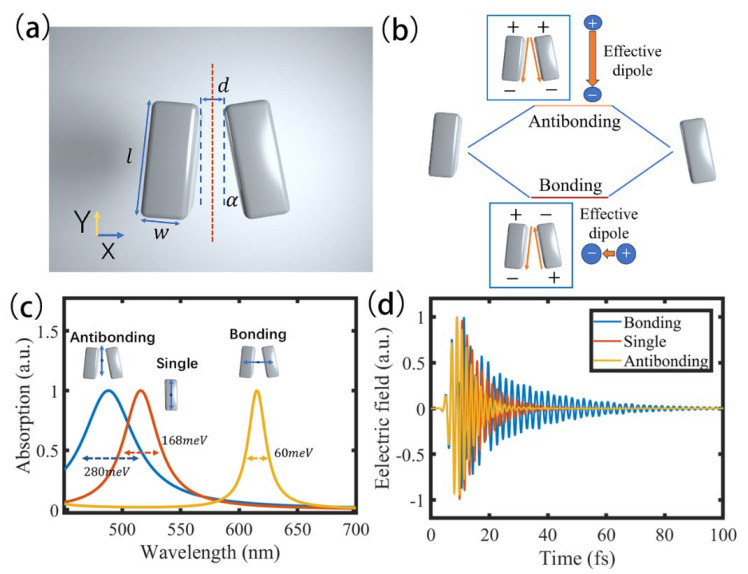
(**a**) Geometry of the silver NCD (80 × 30 × 30 nm^3^) featuring a 6 nm gap and a 5° vertical tilt. (**b**) Schematic illustrations of the bonding and antibonding plasmonic modes in the silver NCD. The bonding mode exhibits an antisymmetric charge distribution, while the antibonding mode is symmetric. (**c**) Absorption spectra of the bonding and antibonding modes of the NCD, with dimensions identical to those in panel (**a**), as well as the longitudinal resonance mode of a single nanocuboid extracted from the dimer. The blue arrows indicate the excitation schemes for the different modes. (**d**) Time-domain decay dynamics of the bonding/antibonding mode of the NCD, and the longitudinal resonance mode of the single nanocuboid, corresponding to the reconance modes in panel (**c**).

In this section, we describe the designed silver NCD that serves as the building block for the strong-coupling system and analyze its resonance modes. Figure 1a depicts the structural configuration of the silver NCD. Each nanocuboid has dimensions of 80 nm in length (l=80 nm) and 30 nm in both width and height (w=h=30 nm). The two nanocuboids are positioned side-by-side with a 6 nm gap, and each is tilted by 5° (α=5°) relative to the vertical axis. Compared to individual metallic nanoparticles, NCDs support a richer set of plasmonic resonance modes and offer additional tunable degrees of freedom, providing a more versatile platform for constructing strong coupling systems. In experiments, NCD samples can be fabricated using electron-beam lithography (EBL) [39] or DNA-assisted nanoparticle self-assembly techniques [21].

Figure 1b shows schematic illustrations of the bonding and antibonding modes that arise from the hybridization of the two nanocuboids in the dimer. In the bonding mode, the surface charge distribution is antisymmetric along the vertical axis, whereas in the antibonding mode it is symmetric. These distinct charge-distribution symmetries dictate the radiative characteristics and excitation conditions of the two modes. For small tilt angles (α<45°), the bonding mode exhibits a reduced effective dipole moment relative to that of a single nanocuboid viewed from the far field, leading to significantly suppressed radiative intensity [52,56]. This subradiant nature minimizes radiative losses while maintaining a small mode volume. The effective dipole moment of the bonding mode is oriented along the x-axis, making it excitable only by x-polarized light. By contrast, the antibonding mode possesses a larger effective dipole moment than a single nanocuboid and, thus, radiates more efficiently, incurring higher radiative losses [57]. When the tilt angle approaches 0°, the bonding mode becomes a completely dark mode that cannot be excited or detected in the far field. Therefore, a small but finite tilt angle was chosen in this work to balance reduced radiative loss with accessibility in far field.

Figure 1c shows the absorption spectra of the bonding mode of the silver NCD, the antibonding mode of the silver NCD, and the longitudinal resonance mode of a single nanocuboid calculated by FDTD simulation. In the simulation, the permittivities of silver are taken from Johnson and Christy’s data [58]. The bonding and antibonding modes are excited by x- and y-polarized plane waves, respectively, while the longitudinal resonance mode of a single nanocuboid is excited by a plane wave polarized along its long axis, as indicated by the blue arrows in Figure 1c. As revealed by the absorption spectra, the bonding mode, resonating at 614 nm, has a much narrower linewidth (60 meV) than both the antibonding mode (280 meV) and the longitudinal resonance mode of an individual nanocuboid (168 meV). Figure 1d further compares the temporal evolution of the electric field intensity for the different modes. The bonding mode clearly exhibits the slowest energy decay rate among them.

According to the criterion for strong coupling, the coupling strength must exceed the total loss rate of the system; moreover, for the same coupling strength, smaller losses correspond to longer coherence times. Therefore, low-loss plasmonic structures offer a significant advantage for studying strong coupling. Unlike conventional single-particle dipole modes, which efficiently couple to the far field and suffer significant radiative damping (usually over 100 meV) [33,34,35,59], the bonding mode in the dimer configuration minimizes radiative leakage through destructive interference between individual dipoles, resulting in a low-loss plasmonic platform well-suited for strong coupling. In addition, the dimer geometry offers a new degree of control—tilt angle of the nanocuboids—that allows further modulation of the resonance loss and, thus, the resulting hybridized states. Consequently, the silver NCD offers a low-loss and highly tunable platform for deeper exploration of plasmon-exciton interaction in two-dimensional materials.

## 3. Results and Discussion

### 3.1. NCD-Monolayer WS_2_ Strong Coupling System

**Figure 2:**
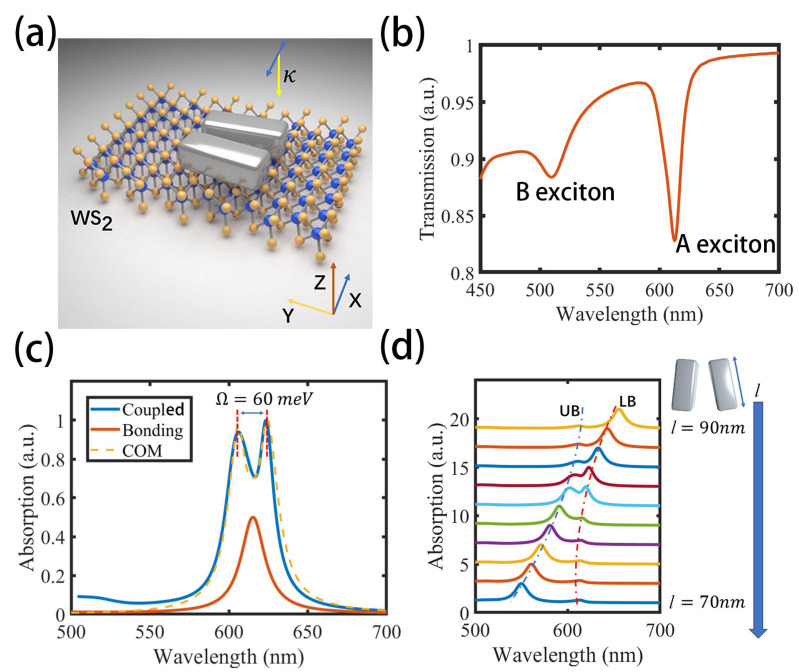
(**a**) Schematic illustration of the hybrid system composed of a silver NCD and a monolayer WS_2_. A normally incident plane wave propagating along the z-axis, with polarization along the x-axis (blue arrow), selectively excites the bonding mode of the dimer. (**b**) Simulated transmission spectrum of the monolayer WS_2_. (**c**) Simulated absorption spectra of the coupling system (blue line) and the uncoupled bonding mode (red line), and the fitting result using COM (yellow dashed line). In the simulation, the dimensions of the NCD are kept consistent with those shown in Figure 1a. (**d**) Evolution of the absorption spectra as a function of nanocuboid length, calculated by FDTD. l, the nanocuboid length gradually decreases from top to bottom to adjust plasmon energy, while other size parameters remain unchanged.

Next, we investigate the construction of the NCD-2D-material strong coupling platform. In this work, tungsten disulfide (WS_2_), a representative transition-metal dichalcogenide [35,39], was selected as the excitonic component. The schematic of the hybrid structure is shown in Figure 2a. A monolayer WS_2_ with a thickness of approximately 0.7 nm was positioned beneath the silver NCD. A normally incident plane wave, propagating along the z-axis and polarized along the x-axis, was employed to selectively excite the bonding plasmonic mode of the dimer. In the FDTD simulations, the dielectric function of WS_2_ was modeled as a superposition of multiple Lorentz oscillators: $\varepsilon \left(E\right)=\varepsilon_{B}-\sum_{j=1}^{M}f_{j}E_{0j}^{2}/\left(E^{2}-E_{0j}^{2}+i\gamma_{0j}E\right)$ [45,60], where $\varepsilon_{B}$ represents the background permittivity, E is the photon energy, and $E_{0j}$, $\gamma_{0j},$ and $f_{j}$ are the resonance energy, damping rate, and oscillator strength of the j-th oscillator, respectively. M is total number of Lorentz oscillators. The parameters used in the simulation are extracted from experimental measurements [61]. The simulated transmission spectrum of the monolayer WS_2_ (shown in Figure 2b) agrees well with experimental data, displaying two pronounced excitonic resonances: the A exciton at 614 nm and the B exciton at 518 nm. The ultranarrow linewidth of the A exciton ($\gamma_{ex}=28\;meV$) offers a distinct advantage for realizing strong light–matter interactions. Consequently, the A exciton was employed as the excitonic mode in the strong coupling configuration. The absorption spectrum of the coupled system (shown in Figure 2c, blue line) clearly reveals the strong interaction between the A exciton in the monolayer WS_2_ and the bonding plasmon mode of the NCD. Relative to the uncoupled bonding mode resonance (shown in Figure 2c, red line), the hybrid spectrum exhibits a pronounced Rabi splitting of Ω=60 meV. According to the strong coupling criterion—that the Rabi splitting exceeds the average linewidth of the system$\;(\Omega >\frac{\gamma_{ex}\;+\;\gamma_{pl}}{2}=44\;meV)$—this hybrid platform operates in the strong coupling regime.

To further elucidate the coupling dynamics, the system is described using a coupled COM, which treats the plasmonic mode of the dimer and the excitonic resonance of WS_2_ as two damped harmonic oscillators coupled with a strength g. The equations of motion can be expressed as:

(1) $$\ddot{a}\left(t\right)+\gamma_{pl}\dot{a}\left(t\right)+\omega_{pl}^{2}a\left(t\right)+gb\left(t\right)=f_{pl}\left(t\right)\;\ddot{b}\left(t\right)+\gamma_{ex}\dot{b}\left(t\right)+\omega_{ex}^{2}b\left(t\right)+ga\left(t\right)=f_{ex}\left(t\right).$$

Here, $a\left(t\right)\;$and $b\left(t\right)$ denote the generalized coordinates (amplitudes) of the plasmonic and excitonic modes, respectively; $\omega_{pl}$ and $\omega_{ex}$ are the uncoupled resonance frequencies. $\gamma_{pl}$ and $\gamma_{ex}$ represent the damping rates. g is the coupling strength, and $F_{pl}(t)/F_{ex}(t)$ describe the external driving term acting on the plasmonic and excitonic oscillators. Under the assumption of time-harmonic conditions, the corresponding parameters can be expressed as $a\left(t\right)=Ae^{-iwt},b\left(t\right)=Be^{-iwt}$, $f_{pl}\left(t\right)=F_{pl}e^{-iwt},f_{ex}\left(t\right)=F_{ex}e^{-iwt},$ while $A,\;B,{\;F}_{pl}{,F}_{ex}$ are the complex amplitudes of the corresponding physical quantity. Substituting the relevant parameters into Equation (1), we can obtain

(2) $$\left(\begin{array}{cc} \omega_{pl}^{2}-\omega^{2}-i\gamma_{pl}\omega & g\\ g & \omega_{ex}^{2}-\omega^{2}-i\gamma_{ex}\omega \end{array}\right)\left(\begin{array}{c} A\\ B \end{array}\right)=\left(\begin{array}{c} F_{pl}\\ F_{ex} \end{array}\right).$$

By solving the equation, we can obtain:

(3) $$A\left(\omega \right)=\frac{F_{pl}\left(\omega_{ex}^{2}-\omega^{2}-i\gamma_{ex}\omega \right)-gF_{ex}}{{(\omega }_{pl}^{2}-\omega^{2}+i\gamma_{pl}\omega )(\omega_{ex}^{2}-\omega^{2}-i\gamma_{ex}\omega )-g^{2}}\;\;B\left(\omega \right)=\frac{F_{ex}\left(\omega_{pl}^{2}-\omega^{2}-i\gamma_{pl}\omega \right)-gF_{pl}}{{(\omega }_{pl}^{2}-\omega^{2}+i\gamma_{pl}\omega )(\omega_{ex}^{2}-\omega^{2}-i\gamma_{ex}\omega )-g^{2}}.\;$$

Since the excitonic material responds much more weakly to external optical fields compared to plasmons, the term $F_{ex}$ can be neglected in the equations. Therefore, the total polarization of the coupled system can be expressed as $P=(\frac{Q_{pl}\left(\omega_{ex}^{2}\;-\;\omega^{2}\;-\;i\gamma_{ex}\omega \right)\;-\;Q_{ex}g}{{(\omega }_{pl}^{2}\;-\;\omega^{2}\;+\;i\gamma_{pl}\omega )(\omega_{ex}^{2}\;-{\;\omega }^{2}\;-\;i\gamma_{ex}\omega )\;-{\;g}^{2}})F_{pl}$, where $Q_{pl},\;Q_{ex}$ are the equivalent charge quantities of the plasmon oscillator and exciton oscillator. $\alpha =\frac{Q_{pl}\left(\omega_{ex}^{2}\;-{\;\omega }^{2}\;-\;i\gamma_{ex}\omega \right)\;-\;Q_{ex}g}{{(\omega }_{pl}^{2}\;-\;\omega^{2}\;+\;i\gamma_{pl}\omega )(\omega_{ex}^{2}\;-\;\omega^{2}\;-\;i\gamma_{ex}\omega )\;-\;g^{2}}\;$ corresponds to the equivalent polarization rate of the system; therefore, the absorption cross section can be given by the following [62]: $\delta \left(\omega \right)\;\propto \;Im\left(\alpha \right)$. In our system, not all excitons in WS_2_ couple to the plasmonic mode; uncoupled excitons still contribute to the overall absorption spectrum. Therefore, to more accurately fit the simulation results, an additional Lorentzian absorption term $c/{(\omega }_{ex}^{2}-\omega^{2}-i\gamma_{ex}\omega )$ was incorporated into the total equivalent polarization rate α, where c is the fitting parameters to reflect the intensity of unbound excitons. The COM fitting results are shown in Figure 2c as yellow dashed lines, exhibiting excellent agreement with the FDTD simulations.

Another hallmark of the strong coupling regime is the anticrossing behavior of the two hybridized branches—upper band (UB) and lower band (LB)—as the detuning is tuned. Figure 2d depicts the FDTD-calculated evolution picture of the coupling system’s absorption spectra as a function of the nanocuboid length l (90 nm to 70 nm). A pronounced anticrossing behavior emerges when the detuning approaches zero (l=80 nm), unequivocally indicating that the system operates within the strong coupling regime.

In the near-resonant limit ($\omega_{pl}\approx \omega_{ex}\approx \omega_{0}$), the eigenenergies of the coupled system can be derived from Equation (2), which satisfy the following eigenvalue equation:

(4) $$\left(\begin{array}{cc} E_{pl}-\frac{i\gamma_{pl}}{2} & G\\ G & E_{ex}-\frac{i\gamma_{ex}}{2} \end{array}\right)\left(\begin{array}{c} A\\ B \end{array}\right)=E\left(\begin{array}{c} A\\ B \end{array}\right).$$

**Figure 3:**
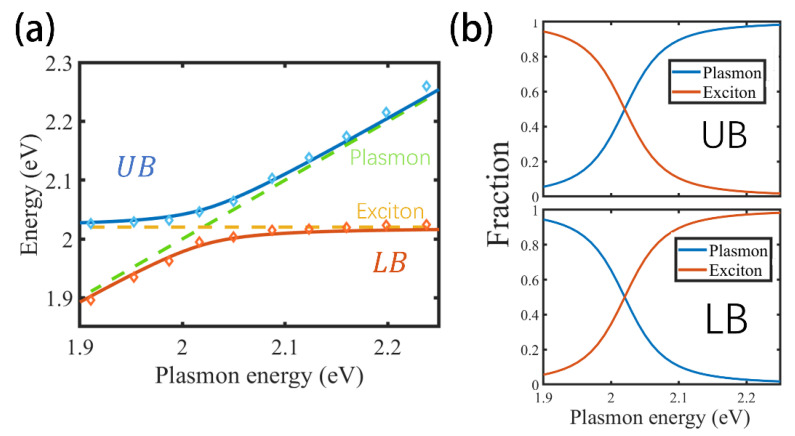
(**a**) Dispersion relations of the hybrid branches calculated using the COM. The blue and yellow diamond markers denote the resonant energies extracted from the simulated absorption spectra. The yellow dotted line and the green dotted line represent the uncoupled plasmon and exciton energies. (**b**) Fraction of the plasmonic and excitonic components in the UB and LB as a function of plasmon energy.

Here, $E_{pl}$, $E_{ex}$ correspond to the energy of the plasmon and the exciton, respectively. E corresponds to the energy of the coupling system. $G=\frac{g}{2\omega_{0}}$ is the normalized coupling strength. By solving the equations, the complex eigenenergies are given by:

(5) $$E_{\pm }=\frac{E_{pl}+E_{ex}}{2}-i\frac{\gamma_{pl}+\gamma_{ex}}{4}\pm \frac{1}{2}\sqrt{{4G}^{2}+{\left(∆+i\frac{\gamma_{pl}-\gamma_{ex}}{2}\right)}^{2}},$$

where $∆=E_{pl}-E_{ex}$ is the energy detuning. At resonance $E_{pl}=E_{ex}$, the Rabi splitting is $\Omega =\sqrt{{4G}^{2}-{\left(\frac{\gamma_{pl}-{\;\gamma }_{ex}}{2}\right)}^{2}}$. From the measured splitting of 60 meV and $\gamma_{pl}=60\;meV,\gamma_{ex}=28\;meV$, we obtain G≈31 meV. Figure 3a presents the dispersion of the hybrid branches calculated using the COM, where the upper branch (UB) corresponds to $E_{+}$ (blue solid line) and the lower branch (LB) corresponds to $E_{-}$ (red solid line). The blue and yellow diamond markers represent the resonant energies of the hybrid branches extracted from the simulated absorption spectra. The dispersion curves clearly reveal the characteristic anticrossing behavior of the hybrid branches, further confirming that the system step into the strong coupling regime. By solving the eigenvalue Equation (3), the eigenvectors$\;{\left(\begin{array}{cc} A & B \end{array}\right)}^{T}$ of the coupled system can be obtained. Normalizing these eigenvectors ($A^{2}+B^{2}=1$) yields the fractional contributions of the plasmonic$\;(A^{2})$ and excitonic$\;(B^{2})$ components—also known as the Hopfield coefficients—for each hybrid mode. Figure 3b presents the fractions of the upper branch (UB) and lower branch (LB) under various plasmon energies. As the plasmon energy increases, the LB gradually transitions from plasmon-like character to an exciton-like character, whereas the UB exhibits the opposite trend. At zero detuning, the plasmonic and excitonic components each contribute 50% to both hybrid branches, reflecting the mixing hybridization characteristic of strong coupling.

Table 1 summarizes representative studies on strong coupling between plasmonic structures and TMDCs. In general, the criterion for strong coupling requires the Rabi splitting to exceed the total loss of the coupled modes. To more clearly describe this requirement, we introduce a critical coupling strength, defined as the condition where the Rabi splitting equals the system loss ($\Omega =\frac{\gamma_{ex}\;+\;\gamma_{pl}}{2}$). Based on COM, this critical coupling strength can be explicitly expressed: $G_{c}=\sqrt{\frac{\gamma_{ex}^{2}\;+{\;\gamma }_{pl}^{2}}{8}}$, and the corresponding values for different works are also listed in Table 1. As shown in the comparison, our nanocuboid dimer system exhibits substantially lower plasmonic losses compared to previously reported structures. As a direct consequence, the associated critical coupling strength is markedly reduced, thereby facilitating access to the strong coupling regime even at moderate interaction strengths. In our system, the ratio of the coupling strength to the critical coupling strength also exceeds that of the vast majority of previously reported works. This further confirms that our nanocuboid dimer platform not only relaxes the requirements for entering the strong coupling regime but also provides a highly efficient pathway for realizing plasmon–TMDC hybrid strong coupling. It is worth noting that in Ref. [46], S. Wang et al. achieved plasmonic losses and critical coupling strengths comparable to those in our work. However, their approach relied on collective surface plasmonic lattice resonances to realize ultralow losses, which is fundamentally different from the localized mode employed here. Such lattice resonances require large-area nanoparticle arrays for support, and their field confinement is considerably weaker, making them less suitable for investigating strong coupling in the few-exciton situation and limiting their application in local hybrid devices.

### 3.2. Coupled System Regulation Schemes

Finally, we discuss the tunability of the NCD–WS_2_ coupled system. According to the fundamental principles of strong coupling, the coupling state of a system is determined by two key parameters: the coupling strength and the system loss. Accordingly, two strategies can be envisioned for tuning such systems—modulating the coupling strength or modulating the loss. However, most previous works have focused almost exclusively on tuning the coupling strength, for example, by varying the number of TMDC layers [40,45] or the concentration of dye molecules [48,49], while studies exploiting loss modulation remain rare. Importantly, coupling strength reflects the rate of coherent energy exchange, whereas loss represents the rate of energy dissipation; these are two distinct aspects of system performance. Therefore, achieving both coupling-strength modulation and loss modulation within the same platform enriches the available control schemes and offers versatile pathways to adapt strong-coupling systems for different application scenarios. In our work, we demonstrate both approaches by proposing two distinct strategies for regulating the hybrid system.

**Figure 4:**
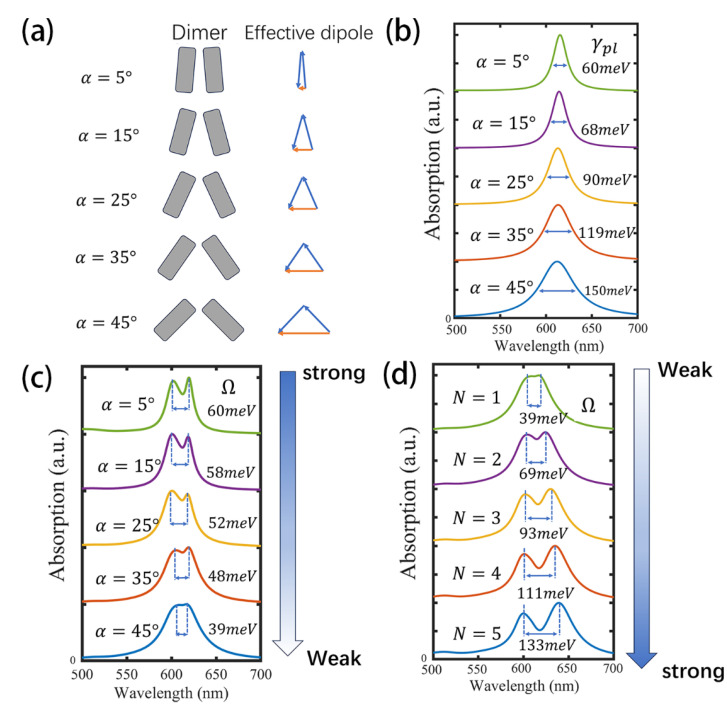
(**a**) Schematic of tilt angle tuning of NCDs and the effective dipole moments of the supported bonding modes, illustrating how the effective dipole moment of the bonding mode evolves with tilt angle. In the simulation, the dimensions of nanocuboid are 80 × 30 × 30 nm^3^, and the gap is fixed at 6 nm. (**b**) Absorption spectra of the NCD as a function of tilt angle. (**c**) Absorption spectra of the NCD–monolayer WS_2_ coupled system for different tilt angles. (**d**) Absorption spectra of coupled systems with varying numbers of WS_2_ layers and a fixed 45° nanocuboid tilt angle.

First, by adjusting the tilt angle of the nanocuboids from 5° to 45°, the effective dipole moment of the bonding mode can be gradually increased as shown in Figure 4a, thereby enabling precise control over its radiative loss [64]. Figure 4b presents the absorption spectra of the NCD as a function of tilt angle, showing that the loss of the bonding mode ($\gamma_{pl}$) can be tuned from 60 to 150 meV when the angle is tuned from 5° to 45°. To maintain resonance between the plasmonic mode and the WS_2_ exciton at 614 nm, the length of the nanocuboids was slightly adjusted at each tilt angle.

The plasmonic loss plays a key role in determining the coupling regime of the hybrid system. First, according to the strong coupling criterion—that the Rabi splitting must exceed the average loss of the coupled system, i.e., $\Omega \geq \frac{\gamma_{pl}\;+\;\gamma_{ex}}{2}$—an increase in loss necessitates a higher coupling strength to reach the strong coupling regime. Second, based on the COM, the magnitude of the Rabi splitting can be expressed as a function of the loss difference between the plasmon and the exciton: $\Omega =\sqrt{g^{2}-{\left(\frac{\gamma_{pl}-\gamma_{ex}}{2}\right)}^{2}}$. As this loss difference $\frac{\gamma_{pl}-\gamma_{ex}}{2}$ increases, the effective Rabi splitting decreases. Therefore, tuning the plasmonic loss serves as an effective strategy for controlling the coupling state of the system. Figure 4c shows the absorption spectra of the dimer–monolayer WS_2_ coupled system for different tilt angles. As tilt angle increases and the loss becomes larger, the Rabi splitting becomes progressively masked by linewidth broadening, signifying a transition from the strong to the weak coupling regime. At a tilt angle of 5°, the Rabi splitting of the coupled system Ω=60 meV exceeds the average loss $\frac{\gamma_{pl}+\gamma_{ex}}{2}=44$ meV, indicating that the system operates in the strong coupling regime, whereas at 45°, the Rabi splitting Ω=39 meV falls below the average loss $\frac{\gamma_{pl}+\gamma_{ex}}{2}=89$ meV, indicating that the system operates in the weak coupling regime. In the weak and strong coupling regimes, plasmon–exciton interactions exhibit distinct applications. In the weak coupling regime, they are closely associated with the Purcell effect, enabling enhanced spontaneous emission rates and improved light extraction, which are highly relevant for light-emitting devices and single-photon sources [65,66]. By contrast, in the strong coupling regime, coherent energy exchange between plasmons and excitons leads to the formation of hybrid polaritonic states, which underpin applications such as low-threshold polariton lasing [67], quantum information processing [7], and Bose-Einstein condensation [68].

The second tuning strategy involves adjusting the layer number N of the WS_2_, which can also enable modulation of the coupled system from the weak to the strong coupling regime. In this strategy, the tilt angle of the nanocuboid is fixed at 45°, while the number of WS_2_ layers is gradually increased. Figure 4d presents the absorption spectra of coupled systems incorporating different numbers of WS_2_ layers. As seen from the figure, increasing the number of layers enhances the number of excitons participating in the interaction, leading to a gradual increase in the Rabi splitting and a transition of the coupled system from the weak to the strong coupling regime. 

## 4. Conclusions

In summary, we have designed and numerically demonstrated a low-loss plasmon–exciton strong coupling platform based on a silver NCD and monolayer WS_2_. The dimer supports a subradiant bonding plasmonic mode with a linewidth as narrow as 60 meV, providing a favorable condition for achieving strong coupling at relatively low coupling strengths. FDTD simulations and COM analysis reveal clear Rabi splitting and characteristic anticrossing behavior in the dispersion relations, confirming operation in the strong coupling regime. We also propose and evaluate two effective tuning strategies for the coupled system. The first, based on adjusting the nanocuboid tilt angle, enables control over the bonding mode’s radiative loss, thereby modulating the coupling regime. The second, achieved by varying the number of WS_2_ layers, allows tuning of the coupling strength through the exciton population. Both strategies enable transitions between weak and strong coupling regimes. Our results not only provide a versatile platform for exploring light–matter interactions in plasmonic–2D material hybrids but also offer practical tuning approaches for engineering coupling states, paving the way for applications in nanophotonic devices, quantum optics, and room-temperature polaritonic technologies.

## Figures and Tables

**Table 1:** Studies on strong coupling between plasmonic structures and TMDCs.

Structrues	Plasmonic Loss	Rabi Splitting	Coupling Strength G ^1^	$\mathrm{Critical}\;\mathrm{Coupling}\;\mathrm{Strength}\;G_{c}$ ^1^	$g/g_{c}$
Square dimer [39]	222–252 meV	100.2–128.6 meV	64–75 meV	82–92 meV	<1
Square dimer [55]	248 meV	138 meV	84 meV	91 meV	<1
Nanorod [34]	149 meV	106 meV	58 meV	57 meV	≈1
Bipyramid [33]	110 meV	100 meV	53 meV	42 meV	≈1.25
Nanodisk [47]	170 meV	108 meV	64 meV	61 meV	≈1
Nanotrenches [31]	130 meV	87 meV	48 meV	49 meV	≈1
Nanocube on mirror [59]	220 meV	145 meV	84 meV	80 meV	≈1
Nanoprism-film gap [63]	180 meV	163 meV	87 meV	66 meV	≈1.32
Multi-singular metasurface [45]	380 meV	285 meV (unstrained) 450 meV (strained)	116 meV (unstrained) 240 meV (strained)	135 meV	≈1.22 ≈1.78
Nanoparticle array [46]	54–67 meV	47–100 meV	25–50 meV	22–32 meV	1.14–1.56
NCD (this work)	60 meV	60 meV	31 meV	24 meV	≈1.35

^1^ For ease of comparison, the coupling strengths G reported for the various works and the critical coupling strength $G_{c}$ were recalculated consistently using Equation (4) and $G_{c}=\sqrt{\left(\gamma_{ex}^{2}+\gamma_{pl}^{2}\right)/8}$, and the resulting values are presented in Table 1.

## Data Availability

The data that support the findings of this study are available from the corresponding author upon reasonable request.

## References

[1] Bose R., Cai T., Choudhury K.R., Solomon G.S., Waks E. (2014). All-optical coherent control of vacuum Rabi oscillations. Nat. Photonics.

[2] Nomura M., Kumagai N., Iwamoto S., Ota Y., Arakawa Y. (2010). Laser oscillation in a strongly coupled single-quantum-dot–nanocavity system. Nat. Phys..

[3] Reithmaier J.P., Sek G., Loffler A., Hofmann C., Kuhn S., Reitzenstein S., Keldysh L.V., Kulakovskii V.D., Reinecke T.L., Forchel A. (2004). Strong coupling in a single quantum dot-semiconductor microcavity system. Nature.

[4] McKeever J., Boca A., Boozer A.D., Buck J.R., Kimble H.J. (2003). Experimental realization of a one-atom laser in the regime of strong coupling. Nature.

[5] Imamog¯lu A., Awschalom D.D., Burkard G., DiVincenzo D.P., Loss D., Sherwin M., Small A. (1999). Quantum Information Processing Using Quantum Dot Spins and Cavity QED. Phys. Rev. Lett..

[6] Khitrova G., Gibbs H.M., Kira M., Koch S.W., Scherer A. (2006). Vacuum Rabi splitting in semiconductors. Nat. Phys..

[7] Chang D.E., Vuletić V., Lukin M.D. (2014). Quantum nonlinear optics—Photon by photon. Nat. Photonics.

[8] Liu C., Zhu T.-X., Su M.-X., Ma Y.-Z., Zhou Z.-Q., Li C.-F., Guo G.-C. (2020). On-Demand Quantum Storage of Photonic Qubits in an On-Chip Waveguide. Phys. Rev. Lett..

[9] Aoki T., Dayan B., Wilcut E., Bowen W.P., Parkins A.S., Kippenberg T.J., Vahala K.J., Kimble H.J. (2006). Observation of strong coupling between one atom and a monolithic microresonator. Nature.

[10] Thompson R.J., Rempe G., Kimble H.J. (1992). Observation of normal-mode splitting for an atom in an optical cavity. Phys. Rev. Lett..

[11] Törmä P., Barnes W.L. (2015). Strong coupling between surface plasmon polaritons and emitters: A review. Rep. Prog. Phys..

[12] Chikkaraddy R., de Nijs B., Benz F., Barrow S.J., Scherman O.A., Rosta E., Demetriadou A., Fox P., Hess O., Baumberg J.J. (2016). Single-molecule strong coupling at room temperature in plasmonic nanocavities. Nature.

[13] Li W., Liu R., Li J., Zhong J., Lu Y.-W., Chen H., Wang X.-H. (2023). Highly Efficient Single-Exciton Strong Coupling with Plasmons by Lowering Critical Interaction Strength at an Exceptional Point. Phys. Rev. Lett..

[14] Liu R., Geng M., Ai J., Fan X., Liu Z., Lu Y.-W., Kuang Y., Liu J.-F., Guo L., Wu L. (2024). Deterministic positioning and alignment of a single-molecule exciton in plasmonic nanodimer for strong coupling. Nat. Commun..

[15] Gupta S.N., Bitton O., Neuman T., Esteban R., Chuntonov L., Aizpurua J., Haran G. (2021). Complex plasmon-exciton dynamics revealed through quantum dot light emission in a nanocavity. Nat. Commun..

[16] Zengin G., Wersäll M., Nilsson S., Antosiewicz T.J., Käll M., Shegai T. (2015). Realizing Strong Light-Matter Interactions between Single-Nanoparticle Plasmons and Molecular Excitons at Ambient Conditions. Phys. Rev. Lett..

[17] Chowdhury M.G.R., Hesami L., Khabir K.M., Howard S.R., Rab M.A., Noginova N., Noginov M.A. (2024). Anomalous Dispersion in Reflection and Emission of Dye Molecules Strongly Coupled to Surface Plasmon Polaritons. Nanomaterials.

[18] Cao S., Xing Y., Sun Y., Liu Z., He S. (2022). Strong Coupling between a Single Quantum Emitter and a Plasmonic Nanoantenna on a Metallic Film. Nanomaterials.

[19] Wu F., Guo J., Huang Y., Liang K., Jin L., Li J., Deng X., Jiao R., Liu Y., Zhang J. (2021). Plexcitonic Optical Chirality: Strong Exciton–Plasmon Coupling in Chiral J-Aggregate-Metal Nanoparticle Complexes. ACS Nano.

[20] Wu F., Li N., Ding B., Zhang W. (2023). Plasmon–Exciton Strong Coupling Effects of the Chiral Hybrid Nanostructures Based on the Plexcitonic Born–Kuhn Model. ACS Photonics.

[21] Zhu J., Wu F., Han Z., Shang Y., Liu F., Yu H., Yu L., Li N., Ding B. (2021). Strong Light–Matter Interactions in Chiral Plasmonic–Excitonic Systems Assembled on DNA Origami. Nano Lett..

[22] Cheng Q., Yang J., Sun L., Liu C., Yang G., Tao Y., Sun X., Zhang B., Xu H., Zhang Q. (2023). Tuning the Plexcitonic Optical Chirality Using Discrete Structurally Chiral Plasmonic Nanoparticles. Nano Lett..

[23] Liang X., Liang K., Deng X., He C., Zhou P., Li J., Qin J., Jin L., Yu L. (2024). The Mechanism of Manipulating Chirality and Chiral Sensing Based on Chiral Plexcitons in a Strong-Coupling Regime. Nanomaterials.

[24] Fofang N.T., Grady N.K., Fan Z., Govorov A.O., Halas N.J. (2011). Plexciton Dynamics: Exciton−Plasmon Coupling in a J-Aggregate−Au Nanoshell Complex Provides a Mechanism for Nonlinearity. Nano Lett..

[25] Nan F., Zhang Y.-F., Li X., Zhang X.-T., Li H., Zhang X., Jiang R., Wang J., Zhang W., Zhou L. (2015). Unusual and Tunable One-Photon Nonlinearity in Gold-Dye Plexcitonic Fano Systems. Nano Lett..

[26] Zhang T., Guo Q., Shi Z., Zhang S., Xu H. (2023). Coherent Second Harmonic Generation Enhanced by Coherent Plasmon–Exciton Coupling in Plasmonic Nanocavities. ACS Photonics.

[27] Li J., Deng X., Jin L., Wang Y., Wang T., Liang K., Yu L. (2023). Strong coupling of second harmonic generation scattering spectrum in a diexcitionic nanosystem. Opt Express.

[28] Wang G., Chernikov A., Glazov M.M., Heinz T.F., Marie X., Amand T., Urbaszek B. (2018). Colloquium: Excitons in atomically thin transition metal dichalcogenides. Rev. Mod. Phys..

[29] Hill H.M., Rigosi A.F., Roquelet C., Chernikov A., Berkelbach T.C., Reichman D.R., Hybertsen M.S., Brus L.E., Heinz T.F. (2015). Observation of Excitonic Rydberg States in Monolayer MoS_2_ and WS_2_ by Photoluminescence Excitation Spectroscopy. Nano Lett..

[30] Gonçalves P.A.D., Stenger N., Cox J.D., Mortensen N.A., Xiao S. (2020). Strong Light–Matter Interactions Enabled by Polaritons in Atomically Thin Materials. Adv. Opt. Mater..

[31] Zhou J., Gonçalves P.A.D., Riminucci F., Dhuey S., Barnard E.S., Schwartzberg A., García de Abajo F.J., Weber-Bargioni A. (2024). Probing plexciton emission from 2D materials on gold nanotrenches. Nat. Commun..

[32] Luo C., Li W., Li J., Fu Z., Hu N., Yu Z., Chang W., Li P., Huang X., Liu B. (2025). Room-Temperature Exciton Polaritons in Monolayer WS(2) Enabled by Plasmonic Bound States in the Continuum. Nano Lett..

[33] Stuhrenberg M., Munkhbat B., Baranov D.G., Cuadra J., Yankovich A.B., Antosiewicz T.J., Olsson E., Shegai T. (2018). Strong Light-Matter Coupling between Plasmons in Individual Gold Bi-pyramids and Excitons in Mono- and Multilayer WSe_2_. Nano Lett..

[34] Wen J., Wang H., Wang W., Deng Z., Zhuang C., Zhang Y., Liu F., She J., Chen J., Chen H. (2017). Room-Temperature Strong Light-Matter Interaction with Active Control in Single Plasmonic Nanorod Coupled with Two-Dimensional Atomic Crystals. Nano Lett..

[35] Zhong J., Li J.Y., Liu J., Xiang Y., Feng H., Liu R., Li W., Wang X.H. (2024). Room-Temperature Strong Coupling of Few-Exciton in a Monolayer WS(2) with Plasmon and Dispersion Deviation. Nano Lett..

[36] Yang J., Zhao L., Song Z., Xiao J., Li L., Zhang G., Wang W. (2025). Ultrafast Investigation of the Strong Coupling System between Square Ag Nanohole Array and Monolayer WS_2_. Nano Lett..

[37] Lu Z., Song D., Lin C., Zhang H., Zhang S., Xu H. (2025). Plexciton Photoluminescence in Strongly Coupled 2D Semiconductor-Plasmonic Nanocavity Hybrid. ACS Nano.

[38] Munkhbat B., Baranov D.G., Bisht A., Hoque M.A., Karpiak B., Dash S.P., Shegai T. (2020). Electrical Control of Hybrid Monolayer Tungsten Disulfide-Plasmonic Nanoantenna Light-Matter States at Cryogenic and Room Temperatures. ACS Nano.

[39] Liu L., Tobing L.Y.M., Wu T., Qiang B., Garcia-Vidal F.J., Zhang D.H., Wang Q.J., Luo Y. (2021). Plasmon-induced thermal tuning of few-exciton strong coupling in 2D atomic crystals. Optica.

[40] Kleemann M.E., Chikkaraddy R., Alexeev E.M., Kos D., Carnegie C., Deacon W., de Pury A.C., Grosse C., de Nijs B., Mertens J. (2017). Strong-coupling of WSe_2_ in ultra-compact plasmonic nanocavities at room temperature. Nat. Commun..

[41] Verellen N., Lopez-Tejeira F., Paniagua-Dominguez R., Vercruysse D., Denkova D., Lagae L., Van Dorpe P., Moshchalkov V.V., Sanchez-Gil J.A. (2014). Mode parity-controlled Fano- and Lorentz-like line shapes arising in plasmonic nanorods. Nano Lett..

[42] Wersäll M., Cuadra J., Antosiewicz T.J., Balci S., Shegai T. (2016). Observation of Mode Splitting in Photoluminescence of Individual Plasmonic Nanoparticles Strongly Coupled to Molecular Excitons. Nano Lett..

[43] Wu F., Jiao R., Yu L. (2021). A Semiclassical Model for Plasmon-Exciton Interaction From Weak to Strong Coupling Regime. IEEE Photonics J..

[44] Li C., Lu X., Srivastava A., Storm S.D., Gelfand R., Pelton M., Sukharev M., Harutyunyan H. (2021). Second Harmonic Generation from a Single Plasmonic Nanorod Strongly Coupled to a WSe2 Monolayer. Nano Lett..

[45] Wu T., Wang C., Hu G., Wang Z., Zhao J., Wang Z., Chaykun K., Liu L., Chen M., Li D. (2024). Ultrastrong exciton-plasmon couplings in WS_2_ multilayers synthesized with a random multi-singular metasurface at room temperature. Nat. Commun..

[46] Wang S., Le-Van Q., Vaianella F., Maes B., Eizagirre Barker S., Godiksen R.H., Curto A.G., Gomez Rivas J. (2019). Limits to Strong Coupling of Excitons in Multilayer WS_2_ with Collective Plasmonic Resonances. ACS Photonics.

[47] Geisler M., Cui X., Wang J., Rindzevicius T., Gammelgaard L., Jessen B.S., Gonçalves P.A.D., Todisco F., Bøggild P., Boisen A. (2019). Single-Crystalline Gold Nanodisks on WS_2_ Mono- and Multilayers for Strong Coupling at Room Temperature. ACS Photonics.

[48] Balci S., Kucukoz B., Balci O., Karatay A., Kocabas C., Yaglioglu G. (2016). Tunable Plexcitonic Nanoparticles: A Model System for Studying Plasmon–Exciton Interaction from the Weak to the Ultrastrong Coupling Regime. ACS Photonics.

[49] Liu R., Zhou Z.-K., Yu Y.-C., Zhang T., Wang H., Liu G., Wei Y., Chen H., Wang X.-H. (2017). Strong Light-Matter Interactions in Single Open Plasmonic Nanocavities at the Quantum Optics Limit. Phys. Rev. Lett..

[50] Davis T.J., Gómez D.E., Vernon K.C. (2010). Simple Model for the Hybridization of Surface Plasmon Resonances in Metallic Nanoparticles. Nano Lett..

[51] Wu F., Zhang W. (2025). Plexcitonic dynamics in plasmonic nanorod dimer strongly coupled to a single quantum dot via modulation of dark modes. Opt. Express.

[52] Zhang S., Genov D.A., Wang Y., Liu M., Zhang X. (2008). Plasmon-induced transparency in metamaterials. Phys. Rev. Lett..

[53] Osberg K.D., Harris N., Ozel T., Ku J.C., Schatz G.C., Mirkin C.A. (2014). Systematic study of antibonding modes in gold nanorod dimers and trimers. Nano Lett..

[54] Gao Y., Zhou N., Shi Z., Guo X., Tong L. (2018). Dark dimer mode excitation and strong coupling with a nanorod dipole. Photonics Res..

[55] Liu L., Tobing L.Y.M., Yu X., Tong J., Qiang B., Fernández-Domínguez A.I., Garcia-Vidal F.J., Zhang D.H., Wang Q.J., Luo Y. (2020). Strong Plasmon–Exciton Interactions on Nanoantenna Array–Monolayer WS_2_ Hybrid System. Adv. Opt. Mater..

[56] Jin Y., Wu K., Sheng B., Ma W., Chen Z., Li X. (2023). Plasmonic Bound States in the Continuum to Tailor Exciton Emission of MoTe2. Nanomaterials.

[57] Huang J.-S., Kern J., Geisler P., Weinmann P., Kamp M., Forchel A., Biagioni P., Hecht B. (2010). Mode Imaging and Selection in Strongly Coupled Nanoantennas. Nano Lett..

[58] Johnson P.B., Christy R.W. (1972). Optical Constants of the Noble Metals. Phys. Rev. B.

[59] Han X., Wang K., Xing X., Wang M., Lu P. (2018). Rabi Splitting in a Plasmonic Nanocavity Coupled to a WS2 Monolayer at Room Temperature. ACS Photonics.

[60] Jiang P., Song G., Wang Y., Li C., Wang L., Yu L. (2019). Tunable strong exciton-plasmon-exciton coupling in WS_2_-J-aggregates-plasmonic nanocavity. Opt. Express.

[61] Wang S., Li S., Chervy T., Shalabney A., Azzini S., Orgiu E., Hutchison J.A., Genet C., Samorì P., Ebbesen T.W. (2016). Coherent Coupling of WS_2_ Monolayers with Metallic Photonic Nanostructures at Room Temperature. Nano Lett..

[62] Bohren C.F., Huffman D.R. (1998). Particles Small Compared with the Wavelength. Absorption and Scattering of Light by Small Particles.

[63] Qin J., Chen Y.H., Zhang Z., Zhang Y., Blaikie R.J., Ding B., Qiu M. (2020). Revealing Strong Plasmon-Exciton Coupling between Nanogap Resonators and Two-Dimensional S emiconductors at Ambient Conditions. Phys. Rev. Lett..

[64] Gao Y., Ge J., Sun S., Shen X. (2022). Dark modes governed by translational-symmetry-protected bound states in the continuum in symmetric dimer lattices. Results Phys..

[65] Lodahl P., Mahmoodian S., Stobbe S. (2015). Interfacing single photons and single quantum dots with photonic nanostructures. Rev. Mod. Phys..

[66] Novotny L., Hecht B. (2012). Principles of Nano-Optics.

[67] Ramezani M., Halpin A., Fernández-Domínguez A.I., Feist J., Rodriguez S.R.-K., Garcia-Vidal F.J., Gómez Rivas J. (2016). Plasmon-exciton-polariton lasing. Optica.

[68] Hakala T.K., Moilanen A.J., Väkeväinen A.I., Guo R., Martikainen J.-P., Daskalakis K.S., Rekola H.T., Julku A., Törmä P. (2018). Bose–Einstein condensation in a plasmonic lattice. Nat. Phys..

